# Reactive saccade adaptation boosts orienting of visuospatial attention

**DOI:** 10.1038/s41598-020-70120-z

**Published:** 2020-08-10

**Authors:** Judith Nicolas, Aurélie Bidet-Caulet, Denis Pélisson

**Affiliations:** 1 Integrative Multisensory Perception Action and Cognition Team (ImpAct), INSERM U1028, CNRS UMR5292, Lyon Neuroscience Research Center (CRNL), 69675 Bron Cedex, France; 2 Brain Dynamics and Cognition (Dycog Team), INSERM U1028, CNRS UMR5292, Lyon Neuroscience Research Center (CRNL), 69675 Bron Cedex, France; 3 Present Address: Movement Control and Neuroplasticity Research Group, Department of Kinesiology, KU Leuven, Tervuursevest 101, Box 1501, 3001 Leuven, Belgium

**Keywords:** Attention, Saccades

## Abstract

Attention and saccadic eye movements are critical components of visual perception. Recent studies proposed the hypothesis of a tight coupling between saccadic adaptation (SA) and attention: SA increases the processing speed of unpredictable stimuli, while increased attentional load boosts SA. Moreover, their cortical substrates partially overlap. Here, we investigated for the first time whether this coupling in the reactive/exogenous modality is specific to the orienting system of attention. We studied the effect of adaptation of reactive saccades (RS), elicited by the double-step paradigm, on exogenous orienting, measured using a Posner-like detection paradigm. In 18 healthy subjects, the attentional benefit—the difference in reaction time to targets preceded by informative versus uninformative cues—in a control exposure condition was subtracted from that of each adaptation exposure condition (backward and forward); then, this cue benefit difference was compared between the pre- and post-exposure phases. We found that, the attentional benefit significantly increased for cued-targets presented in the left hemifield after backward adaptation and for cued-targets presented in the right hemifield after forward adaptation. These findings provide strong evidence in humans for a coupling between RS adaptation and attention, possibly through the activation of a common neuronal pool.

## Introduction

Human beings perform up to 200,000 ocular saccades every day. These rapid eye movements are categorized as either reactive saccades (RS) triggered by the sudden appearance of a stimulus, or voluntary saccades (VS) that are intentionally driven^1^. Any saccadic inaccuracy can result in impaired visual perception^2^. Fortunately, saccade accuracy is maintained throughout life thanks to a plasticity-based visuo-oculomotor learning known as saccadic adaptation (SA). SA is elicited by repeated exposure to saccadic errors (for review^3^). For a long time, the neural substrates of SA were thought to be restricted to the cerebellum and brainstem areas (for review^4^). But recent evidence speaks to the involvement of the cerebral cortex in SA. Indeed, disrupting activity in the intraparietal sulcus (IPS) through transcranial magnetic stimulation modulates both VS and RS adaptation^5^. Moreover, VS adaptation modulates BOLD activity of the IPS and of the inferior precentral sulcus (iPrCS), whereas RS adaptation is associated with activation of the temporo-parietal junction (TPJ), area V5 and iPrCS^6^. Cortical involvement in RS adaptation was confirmed by two other fMRI studies^7,8^.

SA is crucial for visual perception by enhancing eye scanning behavior. Importantly, the contribution of SA to vision might also involve visual processes such as visuospatial attention. Visuospatial attention enhances the processing efficiency of visual signals originating from the area of focus, and simultaneously decreases the processing of signals coming from elsewhere^9,10^. Shifting this focus of attention either without or with saccadic eye movements (covert and overt shifts, respectively) allows us to explore our environment. Similar to the two types of saccades (RS and VS), shifts of attention can react to the sudden appearance of a visual stimulus (exogenous) or be intentionally driven (endogenous). Quite interestingly, the cortical substrates of SA and of visuospatial attention partially overlap and show a modality-related segregation: both VS and endogenous shifts involve the IPS whereas RS and exogenous shifts both recruit the rTPJ^6,11^. Whether these shared neural resources suggest that these two processes interact with one another has been tested and supported by recent studies. First, SA can be facilitated by increasing attentional load^12^. Second, and conversely, McFadden et al.^13^ showed that it is possible to adapt the exogenous shift of attention by ‘stepping the attentional target’ during a covert attentional task (eye movements not allowed) and that this adaptive change transfers to saccades. Third, the reaction time to unpredictable visual stimuli presented in the left hemifield decreased following adaptation of leftward RS^14^. Finally, coupling this behavioral assessment with magnetoencephalography revealed that adaptation of leftward RS increased neural excitability, as indexed by an elevated gamma band power, in a right parietal cortex network including the ventral stream of exogenous shifts of attention^15^. However, note that the un-cued visual detection task used in these last two studies did not allow to specifically manipulate orienting of attention.

Other studies also indirectly support the existence of a coupling between saccadic adaptation and attention shifts. These studies have disclosed that attention, as well as other perceptual or motivational modulatory factors of saccade target selection, can influence saccadic adaptation tested concomitantly^16–20^. However, to directly tackle the main question raised here of whether the attention and adaptation processes overlap, we need to demonstrate that stimulating one process in isolation modifies the second process tested immediately after, e.g. that saccadic adaptation directly modifies our abilities of shifting attention.

The present study aimed at providing valuable evidence for the existence of a coupling between SA and exogenous attention as measured by the speed of attention orienting. Based on data reviewed above, we hypothesized that the adaptation of leftward reactive saccades will lead to a facilitated exogenous orienting of attention, as measured in a detection paradigm derived from Posner’s paradigm^9^. While predicting a coupling when inducing backward adaptation (saccade shortening), we will seek for such coupling also when inducing forward adaptation (saccade lengthening), since different mechanisms could underlie these two types of adaptation^3,21^. Last, a comparison between backward and forward SA will help reveal mechanisms behind SA effects on attention, e.g. the processing of error signals which, by definition, point in opposite directions for forward and backward adaptation.

## Materials and methods

### Subjects

The study adheres to the code of ethics of the World Medical Association – Declaration of Helsinki of 2008 and received the approval of the Ethics Committee of INSERM (CEEI-IRB 00003888, n°16-305). Written informed consent was obtained from all twenty-three subjects and they were paid for their participation. Among these, four subjects were excluded because their saccade gain measured in the adapted hemifield did not show the expected decrease (backward exposure, n = 1 subject) or increase (forward exposure, n = 3 subjects) after adaptation. Another subject was excluded because of frequent responses in ‘No-Target trials’ (false alarms > 30%). The 18 remaining subjects included 17 right-handed subjects and 10 females (mean age 26.11 + /− 4.64 *SD*, Standard Deviation). Their vision was normal or corrected-to-normal. Criteria of exclusion were: history of neurological or psychiatric disorders; cognitive disorders preventing the comprehension of the instructions; severe sleep deprivation during the last 24 h; consumption of psychotropic drugs or alcohol during the 24 h preceding the experiment; and, participation in other experiments involving sensorimotor adaptation during the last week. Each subject was pseudo-randomly assigned to one of the six sub-groups, corresponding to the 6 possible orders of testing in the three experimental sessions (within-subject design, see General Design section). The number of subjects was determined from a power analysis performed through the G*Power software^22^ and based on parameters established from the literature (see Power analysis in the Supplementary Methods).

### Apparatus, stimuli and procedure

#### Apparatus

The entire study was carried out in a dimly lit room. Subjects were installed in a comfortable position with the head stabilized by a chin-rest, cheekbone rests, and forehead support; they faced a computer screen (1920 × 1,080 pixels; 53.5 × 34.5 cm; 144 Hz refresh rate) positioned 57 cm from their eyes. The experiment was temporally-based on the 144 Hz refresh rate of the computer display (frame duration approximately 7 ms), therefore all time intervals reported in the following represent multiples of the frame duration and are rounded to the nearest value in milliseconds. The screen background was grey (50% meaning halfway between the minimum luminance and maximum luminance for each gun) for all the tasks carried out in each session. Psychopy, an open-source software, was used for the stimuli presentation and data collection in all tasks^23^. Movements of the right eye were recorded at a frequency of 1,000 Hz using the remote configuration of the EyeLink 1,000 infrared tracker (SR research, Canada). Each task started with the calibration of the eye tracker by asking subjects to fixate on a series of 5 targets displayed near the borders and at the center of the screen.

#### General design

**Figure 1:**
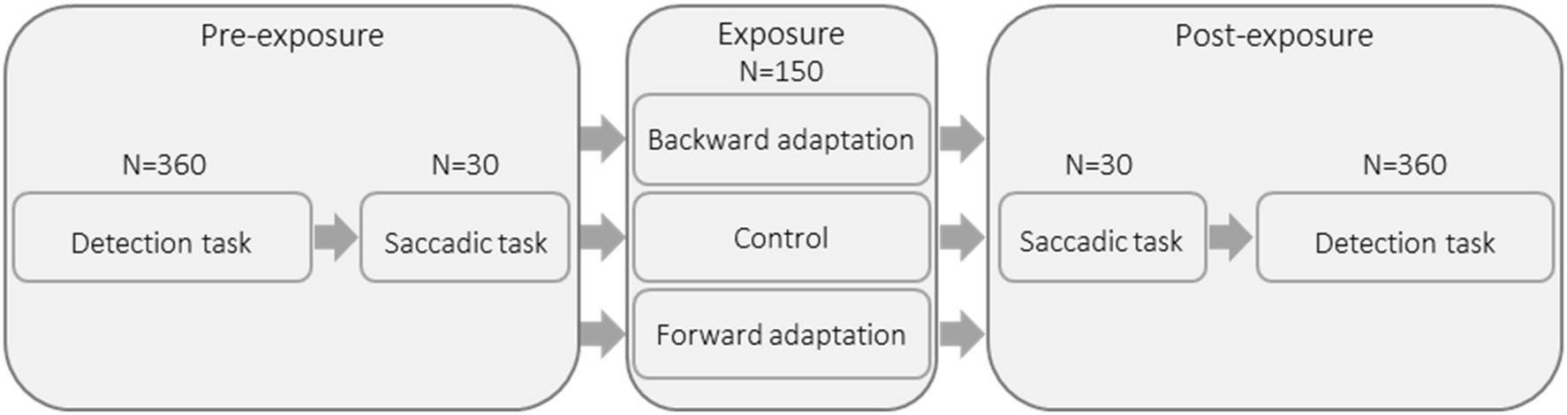
Study general design. Each subject underwent 3 experimental sessions, differing only by the Exposure phase (either backward adaptation, forward adaptation or control). The detection task comprised 3 identical blocks of 120 trials. N = number of trials.

Every subject participated in three experimental sessions (‘backward adaptation’, ‘forward adaptation’ and ‘control’). Each of these sessions consisted of identical pre-exposure and post-exposure phases as well as of a specific exposure phase (Fig. 1). In the backward and forward adaptation conditions, the exposure phase consisted of adaptation of leftward saccades (decrease or increase of saccadic gain, respectively) without adaptation of rightward saccades, whereas during the exposure phase of the control condition, saccades in both directions were not adapted. One fifth of the saccades during the exposure were rightward (randomly inserted) to reinforce the reactive modality with the uncertainty of the target side appearance. The control session provided a baseline measurement of saccades and of visuospatial attention shifts to both the left and right hemifields. It thus allowed, by subtracting this baseline from the same measurements in the two other sessions, the isolation of the specific effects of backward and forward saccadic adaptation taking place in those two sessions. These effects on saccade amplitude and on attention were measured, by comparing between the pre- and post-exposure phases of each session, subjects’ performance in a test saccade task (to verify successful saccadic adaptation) and in a visual detection attentional task. The delay between each session was at least 14 days in order to avoid any retention of saccadic adaptation between sessions, based on a previous study disclosing a significant retention of adaptation up to 5—but not after 11—days after exposure^24^.

#### Saccadic tasks

**Figure 2:**
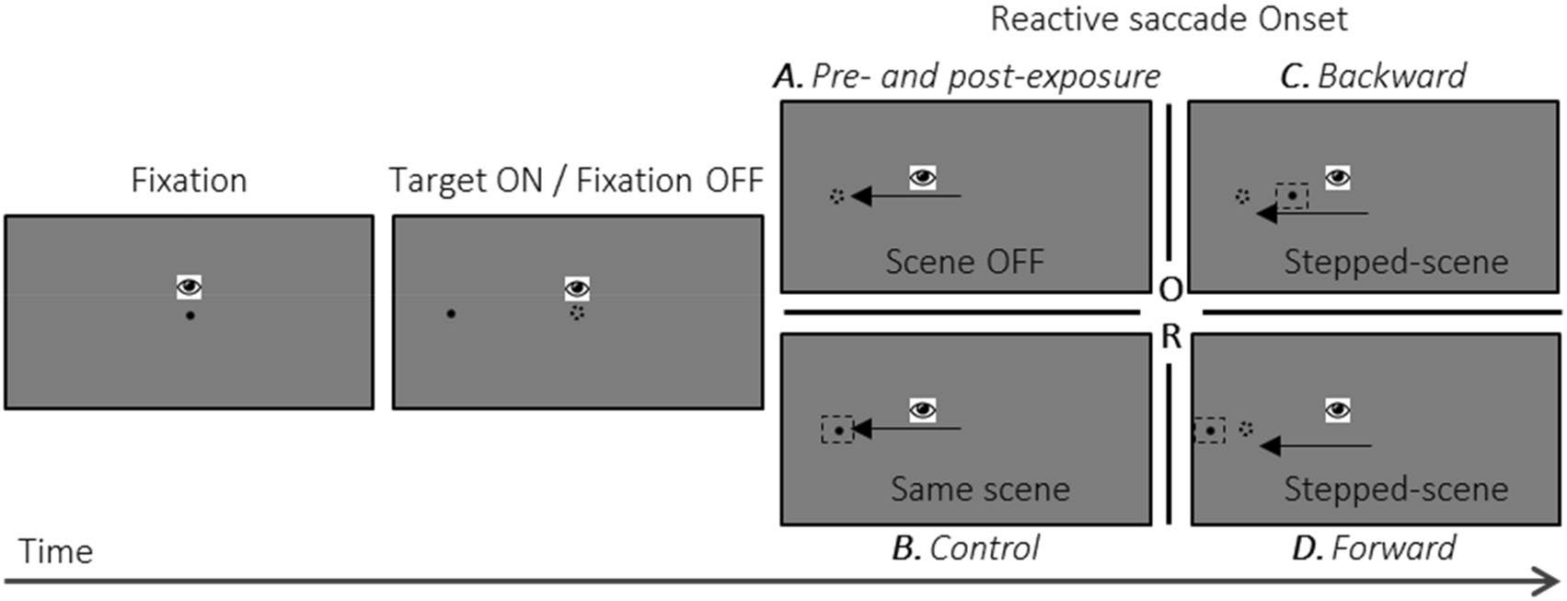
Time-line of trials in the saccadic tasks (not to scale). Subjects were instructed to initiate a saccade as fast and as precise as possible as soon as, after a random fixation period, the central dot is replaced by a peripheral target (11° of eccentricity, to the left in this example). Then, different events occurred upon detection of the reactive saccade, depending on the following conditions. (**A**) In the pre- and post-exposure phases, the visual target was turned off. (**B**) In the control exposure phase, the visual target remained at the same position. (**C**) In the backward adaptation exposure phase, the visual target was shifted 4° backward (final eccentricity: 7°). (**D**) Finally, in the forward adaptation exposure phase, the visual target was shifted 4° forward (final eccentricity: 15°). In all cases, subjects were instructed to keep looking at the peripheral target position for ~ 1 s and then to look back to the center in anticipation of the fixation point re-appearance, using this return period to blink if necessary.

The saccadic adaptation exposure task was performed using a modified version of the double-step paradigm introduced by McLaughlin^25^. This paradigm consists of displacing the visual target while the subject is executing a saccade towards it. As a result of the saccadic suppression phenomenon, this intra-saccadic visual displacement is usually not consciously perceived by subjects and leads to a mismatch between post-saccadic eye fixation and target location which is interpreted by the central nervous system as a saccade aiming error. The sequence of events in adaptation trials is illustrated in Fig. 2B–D. One black fixation dot (+ 50% contrast) of 0.3° of visual angle was displayed at the center of the computer screen. The subject had to fixate on this dot during a pseudo randomized delay of 301–701 ms, after which the central dot disappeared and simultaneously a peripheral target appeared at an eccentricity of 11° and along the horizontal meridian, either to the left or to the right. The side of the peripheral target was randomly assigned between the adapted direction (leftward, 4/5 of the trials) and the opposite un-adapted direction (rightward, 1/5 of the trials). The subject had to initiate a saccade towards the peripheral target and was instructed to be as fast and precise as possible. Correct eye fixation of the central dot was ensured by continuous monitoring of the eyetracker signal (using a circular fixation area with a radius of 3°). The reactive saccade was detected when the eye velocity was higher than 70°/s (for detailed algorithm^26^). When the peripheral target was in the adapted hemifield this event triggered a 4° shift of the visual target (jumping to a 7° or 15° of eccentricity for the backward or forward exposure conditions,Fig. 2C, D respectively), whereas when presented in the un-adapted hemifield, the peripheral target remained at the 11° location. Therefore, the intra-saccadic step was rightward for backward SA and leftward for forward SA. The visual target (shifted or not) remained visible for 805 ms after the detection of the saccade. Subjects were instructed to look at the peripheral target until it turned off. The subjects then had a delay of 1,000 ms to blink and look back to the central dot. Subjects were instructed to blink while looking back at the central fixation dot. The next trial started as soon as fixation around the central dot location was detected.

The saccadic control task, also referred to as the control exposure, was identical to the adaptation tasks except that there was no jump of the visual target in any of the trials (‘Control’ in Fig. 2B), thus both rightward and leftward control trials were identical to adaptation exposure trials when target was presented in the right hemifield.

For all sessions, the exposure phase of 150 trials consisted of 3 blocks of 50 trials (10 with a right target and 40 with a left target). Between each block, the subject was allowed to rest with the head still as long as needed.

The pre- and post-exposure saccadic tasks are illustrated in Fig. 2A. These tasks were identical to the exposure task except that the visual target did not jump but instead was turned off at the initiation of the saccade. Each task consisted of one block of 30 trials (15 with a right target and 15 with a left target, randomly ordered). Comparison between pre- and post-exposure tasks allowed measurement of the exposure after-effect on saccade gain in any given session, and comparison of such after-effect between sessions allowed quantitative assessment of the adaptation strength.

#### Attention task: detection

**Figure 3:**
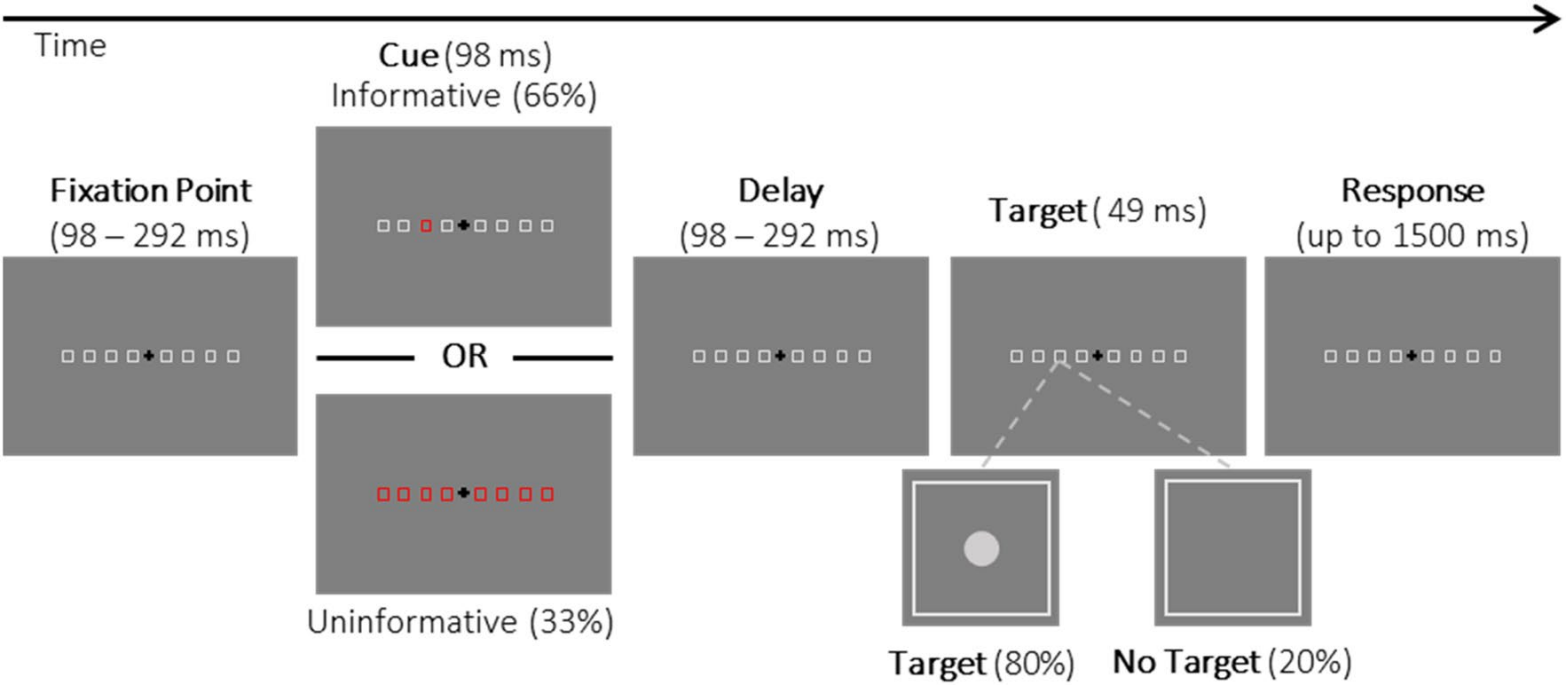
Time-line of a trial in the detection task (not to scale). A central fixation cross and 8 lateral empty placeholders (eccentricity: 3°; 7°; 11°; 15° in each hemifield) were displayed at the beginning of the trial. The placeholders turned red for 98 ms, either indicating the square of the upcoming target (informative cue) or no spatial information (uninformative cue). The target presented after 98 to 292 ms of delay consisted of the brief appearance (49 ms) of a grey dot on the left side (as shown) or the right side (equal probability 50%). Subjects had to respond as fast as possible by pushing a lever when a target was present (Target trials: 80%) or to refrain from responding when there was no target appearing (No- Target trials: 20%).

Covert orienting of exogenous attention was elicited using a variant of the Posner task^9^ designed with general settings (a peripheral cue, and a short Stimulus Onset Asynchrony—SOA) appropriate for shifts of exogenous attention. However, and as mentioned in the discussion, we recognize that the shift of attention elicited in the present study can partially involve endogenous attention^27^, but for the sake of simplification and as it is the orienting component of interest in the present study, we will refer to as exogenous shift of attention. In the present study, contrasting between informative, 100% valid cues (informative trials), and uninformative cues (uninformative trials) allowed us to measure the pure benefit of exogenous orienting whereas, in most exogenous attention studies, the contrast is calculated between valid and invalid cues and thus reflects the cumulated effect of costs and benefits^27,28^. A typical trial is illustrated in Fig. 3. A black fixation cross (+ 50% contrast) subtending 1° of visual angle appeared at the center of the screen at the beginning of the trial and remained visible until the subject’s response. The subject had to keep eye fixation on that location throughout the trial (using a circular fixation area with a radius of 3°). Eight light grey (−15% contrast) empty placeholders (squares of 1.75° of visual angle) were also presented along the horizontal meridian, on the left and on the right, at 3°, 7°, 11°, and 15° of eccentricity. After a pseudo-randomized (98 to 292 ms) delay from the beginning of the trial, the cue – one or all placeholders turning red ([1, −1, −1] in Psychopy RGB color space^23^—appeared for 98 ms. For 80 ‘informative trials’ (out of 120 trials for each block) the cue validly informed the future target location: only one square was highlighted, being predictive of the upcoming target location. In the remaining 40 ‘uninformative’ trials, the cue – all eight placeholders turning red—did not provide any spatial information about the upcoming target. This 2:1 informativeness ratio was meant to reinforce the validity of the cue^29^. In all trials, the cue period was followed by a random time of 98–294 ms after which one grey dot (diameter: 0.3°, −15% contrast) appeared for 49 ms (informative or un-informative trials) or not (‘No-Target’ trials, 16 following informative cues and 8 following uninformative cues). The ‘No-Target’ trials were inserted in order to avoid stereotyped responses to the cue rather than responses to the target per se. The subjects were instructed to detect this grey dot as fast as possible. The maximum duration for detection was 1,500 ms after which the trial ended. Each trial was followed by a blank interval of 1,000 ms. Eye fixation was continuously monitored during the trial and whenever the subject stopped fixating (gaze deviating from the fixation cross by more than 1.5° in any direction), the fixation cross immediately turned red and the trial was aborted. Aborted trials were replayed back during the same block of trials. Subjects were instructed to blink after they responded to the appearance of the target.

The task consisted of 3 blocks of 120 trials each (360 in total): 32 ‘informative—left target’, 32 ‘informative—right target’, 16 ‘uninformative—left target’, 16 ‘uninformative—right target’, and 24 ‘No-Target’ trials (proportionally distributed among trial conditions). The ‘No-Target’ trials were not considered in further analyses. Subjects answered in ‘Target’ trials by pushing away with their index finger a lever-switch in their mid-sagittal axis.

### Data analyses

Data analyses were performed with the open-source software R (The R Core Team, 2013). Any exclusion of a subject due to criteria described in the following paragraphs led to his/her replacement.

#### Saccadic tasks

##### Pre-processing

The eye movement data were analyzed off-line using custom software developed in Matlab (Math Works Inc., Natick, MA, USA). The beginning and end of primary horizontal saccades were identified based on a velocity threshold of 30° s^−1^. Saccadic amplitude was measured as the difference between eye positions 50 ms before the saccade onset and 50 ms after the saccade offset. The main dependent variable was the saccadic gain, computed as the ratio between saccadic amplitude and initial target eccentricity (difference between target position and starting position of the saccade). Saccades with a gain less than 0.5 or outside the mean ± 2 *SD* interval were discarded from further analysis.

##### Statistical analysis

Since saccadic adaptation was critical to test our hypothesis, we excluded from the main analysis subjects who did not show the expected decrease (backward exposure) or increase (forward exposure) of saccade gain in the adapted hemifield. We thus performed, separately for each subject and each hemifield, a unilateral Student t-test comparing the saccadic gain between the pre- and the post-saccadic tasks (*p*-value threshold of 0.05 after FDR-correction for 6 multiple comparisons)^30^. Moreover, we computed the exposure after-effect on saccade gain for each hemifield and each exposure condition as follows:

$$\begin{aligned} & Exposure \; after - effect_{exposure \; of \; interest} \\ & \quad = \frac{{mean \; gain _{post - exposure } - mean \; gain _{pre - exposure } }}{{mean \; gain _{pre - exposure } }} \\ \end{aligned}$$

A negative exposure after-effect reflects a decrease of the saccadic gain between the pre- and the post-exposure phases whereas a positive after-effect reveals an increase.

Finally, to calculate the effect size (Cohen’s *d*) of the exposure after-effect in the backward and the forward adaptation sessions, we computed the mean of the gain for each subject, in the left hemifield for the pre-exposure and the post-exposure phases separately.

#### Attention task

##### Pre-processing

To ensure that the involvement level of all subjects was high, and to exclude those with a too low global performance or too high fluctuations, each session was divided in 24 experimental cells of conditions: 2 cue types (informative or uninformative) × 2 target hemifields (left or right) × 2 phases (pre- or post-exposure) × 3 Blocks (smallest cell = 16 trials). None of the subjects had a number of correct ‘Target’ trials lower than 8 for any of these cells. Outlier RTs of correct trials were excluded using the John Tukey’s method of leveraging the Interquartile Range^31^.

##### Analyses of prerequisites

A significant difference between the informative trials and uninformative trials in the pre-exposure phase was a prerequisite to demonstrate that, at the group level, our detection task readily engaged the orienting of exogenous attention. We also wanted to exclude any influence of the cue-target delay and the hemifield of target appearance on the RT. Thus, a 3-way rmANOVA was performed on detection RT of pre-exposure phases, with Cue type as 2-level factor (informative or uninformative), Cue-target delay as 2-level factor (short or long) and Exposure as 3-level factor (control, backward adaptation, or forward adaptation). The cue-target delays were defined by splitting the eight different cue-target delays into short delays comprised between 98 and 182 ms and long delays comprised between 210 and 294 ms. A main effect of Cue type on RT would satisfy the prerequisite of a significant benefit of orienting attention. Also, the lack of an Exposure effect as well as of interactions between this factor and both the Cue type and the Cue-target delay would allow us to check that, ideally, both the pre-exposure RTs and the pre-exposure RT differences (informative versus uninformative) do not differ between the three conditions and between short and long cue-target delays.

As these prerequisites were met (see Results), we then used as dependent variable the cue benefit which was computed for each subject, each hemifield, each phase (formula below).

$$Cue \; benefit_{exposure \; of \; interest} = \frac{{RT_{Uninformative} - RT_{Informative} }}{{RT_{Informative} }}$$

where *RT*_*Informative*_ and *RT*_*Uninformative*_ represent the median RT of 96 informative trials and 48 uninformative trials, respectively (collapsed across blocks and eccentricity of the same hemifield). Finally, the cue benefit of the control exposure was subtracted from the cue benefit of each adaptation exposure separately (backward or forward).

##### Main statistical analysis

To test our main hypothesis, we performed a 3-way rmANOVA on the cue benefit difference with Exposure (backward and forward), Phase (pre- and post-exposure), and Hemifield (left and right) as within-factors and with subjects as repeated measure. Student t tests were used as post hoc analyses and p-values were FDR corrected (4 comparisons)^30^. The group mean (Fig. 6) was computed as the grand average across all subjects, of the means computed separately for each hemifield (left and right), each phase (pre- and post-exposure) and each experimental condition (backward and forward).

We then assessed the correlation (Pearson's product-moment correlation) between the post-exposure saccadic gain in the adapted hemifield and cue benefit difference in the left hemifield for backward adaptation and in the right hemifield for forward adaptation.

To account for RT variability associated with subject differences and the factors Block and Target eccentricity, we performed a complementary analysis using Generalized linear mixed-effects models (GLMM). The results of this analysis support the results of the main statistical analysis (see supplementary data).

## Results

### Pre- and post-exposure saccadic tasks

**Figure 4:**
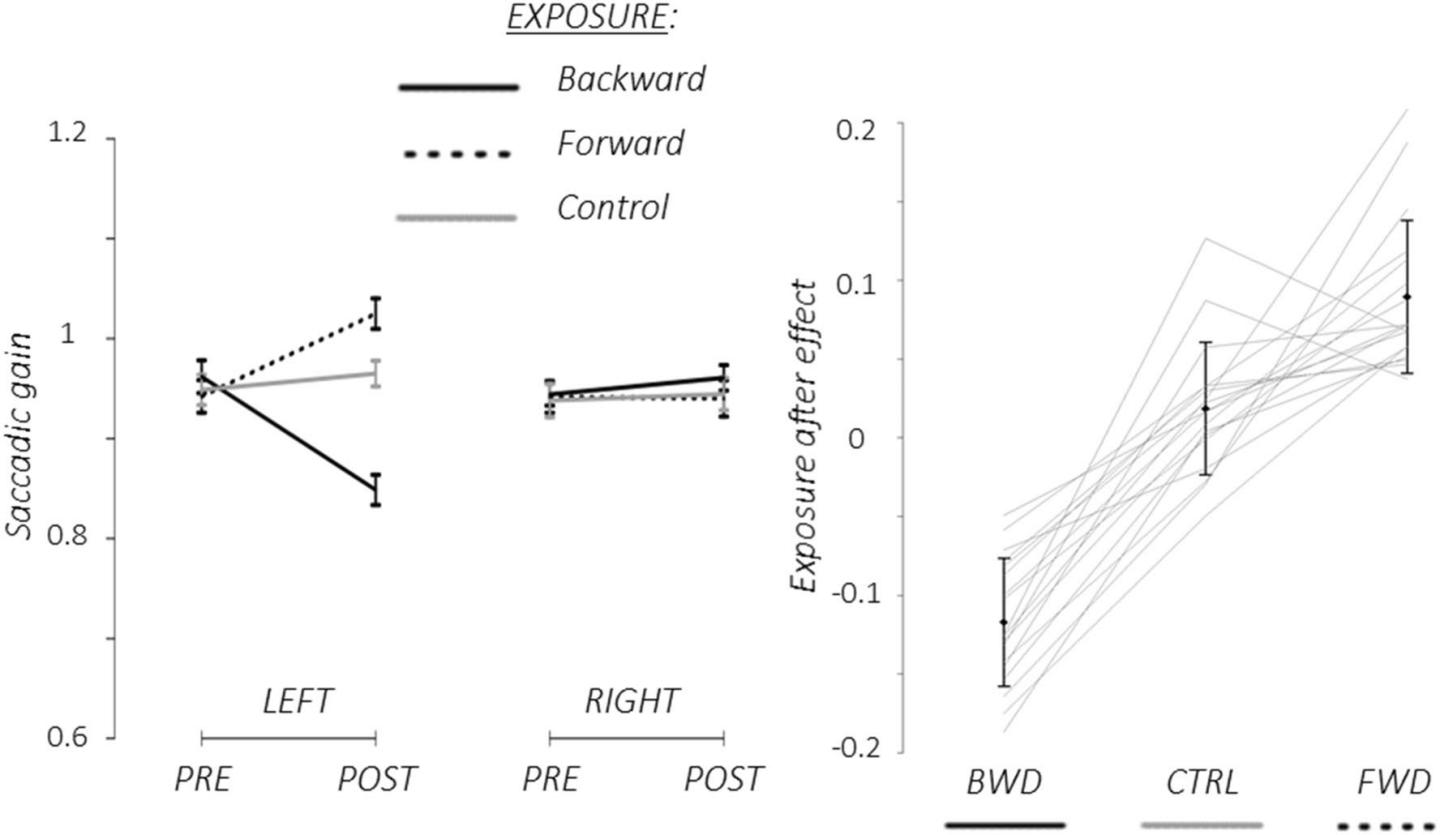
Pre- and Post-exposure saccadic task results. Left panel: Group mean (+/− SD) of saccadic gain. Black lines: backward adaptation exposure; Black dotted: forward adaptation exposure; Grey lines: control exposure. Data shown separately for the adapted (left side) and un-adapted (right side) hemifields. Right panel: Individual exposure after-effects (adapted hemifield). Solid black lines represent group mean (+/− SD) and gray lines stand for individual values.

After rejection of trials following the above mentioned criteria, the average number of trials per condition was 13.8 +/− 1.3 *SD* (total number of trials = 15). The mean saccadic gain in pre- and post-exposure, as well as the individual and mean exposure after-effects, are illustrated in Fig. 4. As they complied with our prerequisites, all subjects showed a significant saccadic gain modulation in the adapted hemifield in the post-exposure as compared to the pre-exposure (decrease after backward exposure, increase after forward exposure), thus exhibiting a significant after-effect due to SA (Fig. 4 right panel). Moreover, as seen in Fig. 4 (left panel), this decrease was not seen in the opposite, un-adapted, hemifield, neither for the backward nor forward exposure. In addition, no gain change in either hemifield took place in the control exposure.

Noteworthy, the magnitude of the effect was different between the backward (Cohen’s *d* = 1.69) and the forward (Cohen’s *d* = 1.23) adaptation, an effect that is well documented in the literature (see for review^3^.

### Attention task

#### Checking prerequisites

After rejection of trials following the above mentioned criteria, the average number of trials per condition was 86.7 + /− 5.2 *SD* for the informative trials (total number of trials = 96) and 44.6 +/− 2 *SD* for the uninformative trials (total number of trials = 48). The average false alarm rate was 8.44% + /− 8.55 *SD*.

**Figure 5:**
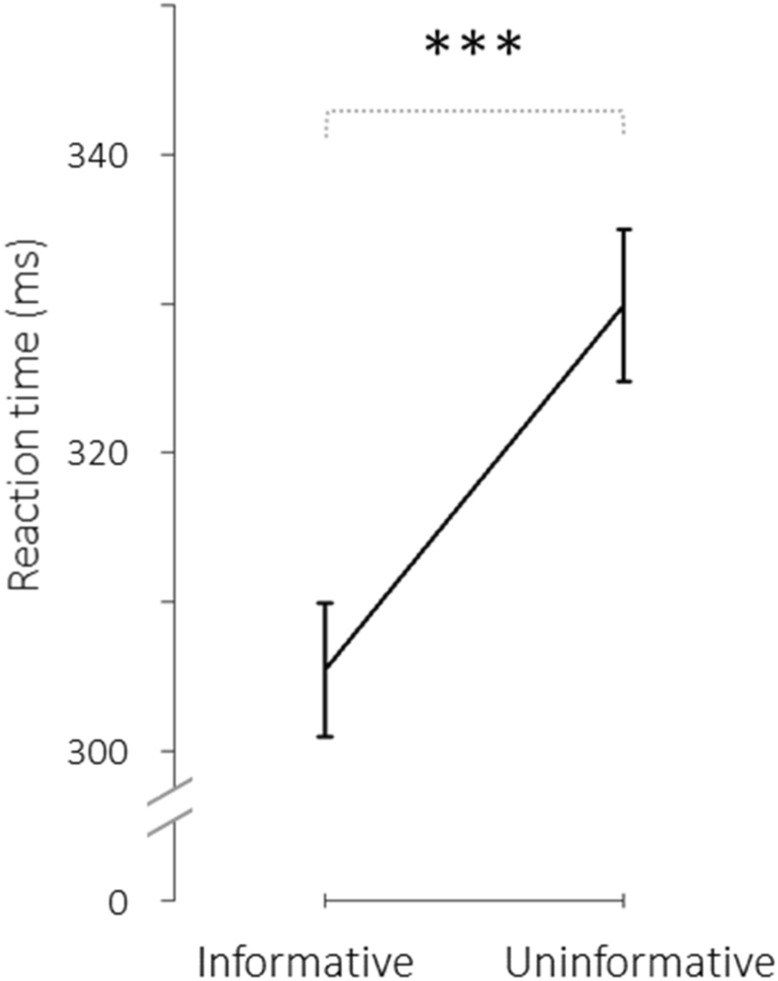
Pre-exposure cue benefit on the reaction times in the attention task. Group mean (+/− SD) of median reaction time (ms) plotted for the informative trials (left) and the uninformative trials (right). ***p value < 0.0001.

The rmAnova testing our prerequisites on the pre-exposure RT revealed a significant main effect of Cue type (partial η^2^ = 0.8; F_(1,17)_ = 66.58; *p* = 2.79e−7), due to a decreased RT in informative trials compared to uninformative trials (Fig. 5). The main effect of Exposure was not significant (F_(2,34)_ = 2.13; *p* = 0.14), nor the main effect of Cue-target delay (F_(1,17)_ = 1.1; *p* = 0.31). Finally, none of the interactions were significant (*p* values > 0.28). Therefore, the Posner-like detection task did engage the exogenous orienting of attention. Moreover, neither the RTs nor the informative versus uninformative RT differences significantly differed between our three sessions before the exposure or between our different cue-target delays. This last observation indicates that the cue benefit collapsed across cue-target-delays in the pre-exposure phase provides a reliable baseline measurement of exogenous attention orienting in all three conditions.

#### Main statistical analysis

**Figure 6:**
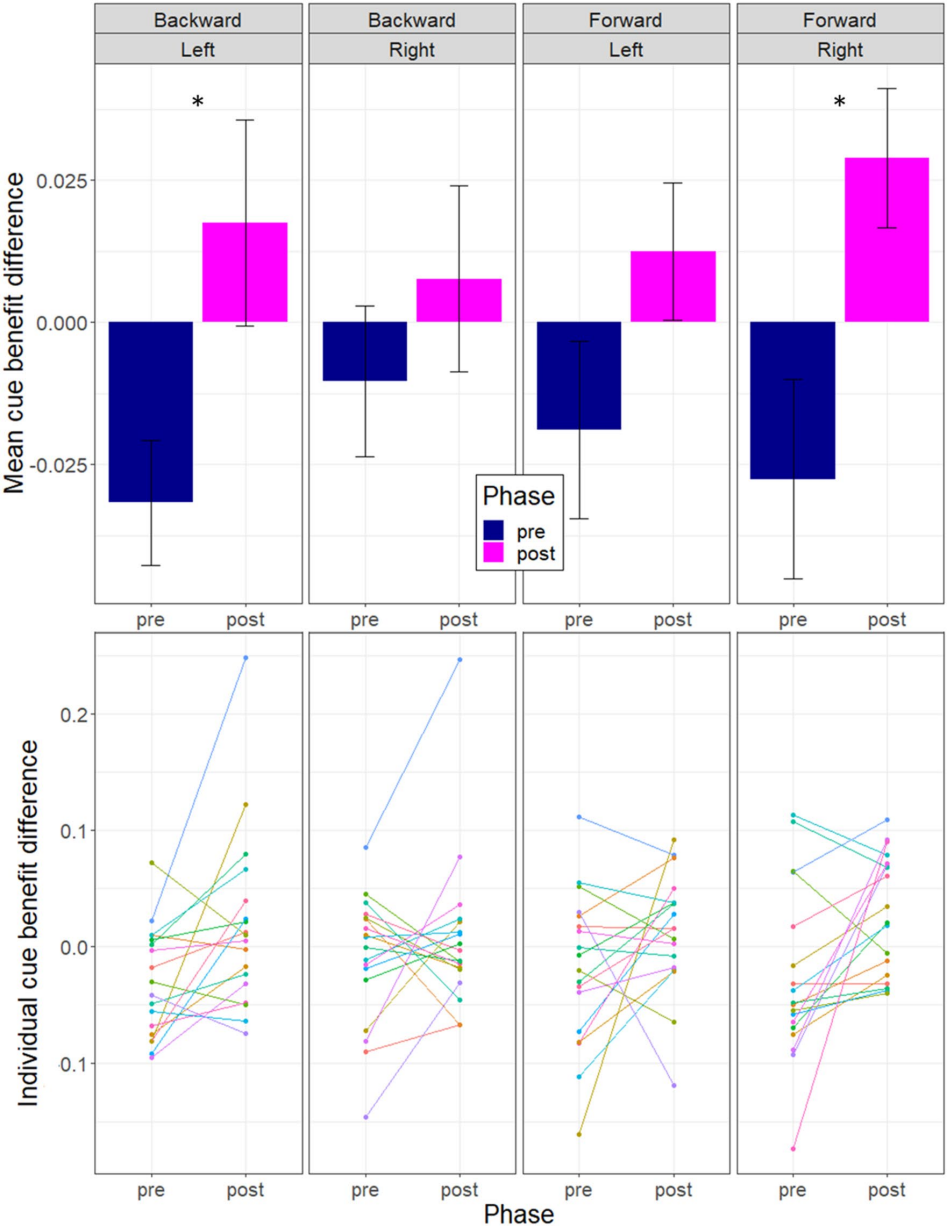
Pre- and Post-exposure cue benefit differences in the attention task. Upper row: Group mean (+/− SE) of cue benefit difference for the pre- and the post-exposure phases in the two hemifields of target presentation (‘Left’, ‘Right’) and in the two adaptation conditions (‘Backward’, ‘Forward’). Blue bars: pre-exposure phases; purple bars: post-exposure phases. Lower row: individual datapoints. *FDR corrected *p* value < 0.05.

The performance in the detection task was evaluated by computing the cue benefit difference (Fig. 6). The 3-way rmANOVA (Exposure x Phase x Hemifield) revealed a significant main effect of phase (partial η^2^ = 0.37; F_(1,17)_ = 10.12; *p* = 0.005) and a significant triple interaction (partial η^2^ = 0.39; F_(1,17)_ = 10.96; *p* = 0.004). The exposure and hemifield main effects were not significant (*p* values > 0.46) and the three double interactions were not significant (*p* values > 0.62). Post hoc analyses revealed that after backward adaptation, the cue benefit difference was increased in the left hemifield but not in the right hemifield (Left: Cohen’s *d* = 0.77; t_(17)_ = −2.71; *p* = 0.015 (0.03 after correction); Right: t_(17)_ = −1.01; *p* = 0.32 (0.32 after correction)). After forward adaptation, the cue benefit difference was increased in the right hemifield but not in the left hemifield (Left: t_(17)_ = 1.55; *p* = 0.14 (0.19 after correction); Right: Cohen’s *d* = 0.88; t_(17)_ = −2.86; *p* = 0.011 (0.03 after correction)).

A relationship between the two adaptation-induced changes revealed above, i.e*.* of cue benefit difference and of saccadic gain, was then tested using a correlation analysis. No significant correlation was revealed between the cue benefit difference and post-exposure saccadic gain in the adapted hemifield, neither for targets in the left hemifield in the backward adaptation session (r_(16)_ = -0.74; *p* = 0.47), nor for targets in the right hemifield in the forward adaptation session, (r_(16)_ = 1.26; *p* = 0.23).

To summarize, the cue benefit difference increased in a spatially-specific way after adaptation of leftward saccades, namely in the left hemifield for backward adaptation and in the right hemifield for forward adaptation. This cue benefit boost in the adaptation sessions seems to be an all-or-none effect since it did not correlate with the exposure after-effect on saccade gain.

## Discussion

The present study demonstrated that leftward SA led to a boost of orienting of attention in the left hemifield following backward adaptation and in the right hemifield following forward adaptation. Our paradigm was designed to elicit exogenous shift of attention. However, the use of cues predicting the spatial location of the upcoming target might have additionally trigger an endogenous orienting of attention, especially for long cue-target delays superior to 300 ms^27^. Note, though, that the lack of significant difference of the cueing effect on median reaction times observed between long and short cue-target delays does not support this hypothesis. Using placeholders and catch trials (20% of no-target trials) might have lengthened the duration of the exogenous peripheral cueing effect^32,33^. Nonetheless, as a follow-up to the present work, one should test whether exogenous attention orienting is modified following VS adaptation and conversely, whether endogenous attention orienting is modified following RS adaptation, to further our knowledge on the modality-specificity of the coupling revealed in the present study.

Our study provides the first demonstration of an effect of SA on covert exogenous attention specifically after adaptation of leftward reactive saccades (RS). The only similar study we are aware of dealt with backward adaptation of both RS and VS, and reported a specific increase of detection performance after adaptation of RS, but not after VS adaptation^14^. This specificity was interpreted in the framework of segregated parieto-frontal systems involved in exogenous and endogenous attention^11^ with a partial overlap of the cortical substrates of adaptation mechanisms for RS and VS, respectively^6^. Note that, contrary to the present study, Habchi et al.’s study^14^ did not address the potential effect of forward SA. In addition, exogenous attention performance was not isolated specifically in a visual-cued detection task but estimated in a speeded discrimination paradigm. Yet, this earlier paradigm likely involved some combination of the three attention systems defined by Petersen and Posner^34^: the alerting, the orienting and, to a lesser extent, the executive systems. Therefore, it was not possible to disentangle whether the accelerated RT was due to a boost of alertness, of attention orienting, or even of motor preparation or decision making. In the present study, we specifically measured exogenous orienting of attention thanks to a detection task where a visual cue (informative or not) was presented peripherally before the target. Noteworthy, both studies underlined the same spatial specificity of the effect of RS backward adaptation on attention, as performance increased in the left (adapted) hemifield. Therefore, the increase of cue benefit we found after SA of leftward RS solidifies Habchi et al.'s.^14^ original findings and their interpretation of a SA-related change of exogenous attention. Other studies have also indirectly supported the existence of a coupling between SA and other types of attention shifts. SA can be induced solely by both a perceptual target^18^ or by a salient visual distractor attracting exogenous attention^17^ flashed in the vicinity of a stationary saccade target. McFadden and colleagues^13^ managed to adapt the exogenous shift of attention by ‘stepping the attentional target’ during a covert attentional task, and showed that this procedure resulted in a change in saccade amplitude. Another study demonstrated that SA efficiency increases with attentional load^12^. These four studies thus suggest that modifications of visuospatial attention can impact saccadic adaptation.

The present study and Habchi et al.’s study^14^ both argue that backward adaptation of leftward RS induces an attentional boosting effect for targets presented in the left, adapted hemifield. These authors interpreted their results as a boost of exogenous orienting (although their design did not actually manipulate orienting of attention) and discussed the specificity of this boost to backward adaptation of leftward -relative to rightward- saccades in the framework of the known dominance of the right hemisphere for attentional processes. This framework is also consistent with the attentional boosting effect observed here after backward adaptation of leftward RS. Moreover, another study from our lab^35^ has investigated the effect of voluntary SA on endogenous visuospatial attention. In this study, we elicited backward adaptation separately in each hemifield, and tested the endogenous orienting using a Posner-like paradigm. The results showed that after adaptation of leftward (and not rightward) VS, the endogenous orienting was boosted for targets in both hemifields. Although addressing different saccade and attention modalities, both studies showed that adaptation of leftward saccades leads to an increase of cue benefit. Accordingly, we argue that in both studies, leftward SA increased neuronal activation in the right posterior parietal cortex (PPC), albeit in different populations of neurons, in turn inducing a boost of leftward attention orienting.

Moreover, the presently demonstrated link between reactive SA and covert exogenous attention provides additional insight into the cortical substrates of these two processes. Thus far, these substrates have been separately delineated by different functional neuroimaging studies, the comparison of which further led to suggest an anatomical overlap in the temporo-parietal junction (see Introduction). However, comparing cortical activation patterns between fMRI studies does not allow to determine the actual anatomical level of overlapping activations (neurons, voxels, or regions of interest). Thus, it remains to be determined whether this anatomical overlap consists of the two functions involving two distinct populations of neurons -albeit intermingled- or involving a single population of neurons, within a circumscribed cortical area. Only in the latter case a temporary change of neuronal excitability induced by one process (e.g. saccadic adaptation) could be expected to transfer to the second process (covert attention). Accordingly, we argue that the link between reactive saccadic adaptation and covert exogenous attention demonstrated in our study favors the hypothesis of these two processes recruiting a common population of neurons within the temporo-parietal junction. Thus, more than just anatomically overlapping, the cortical networks of saccadic adaptation and of exogenous attention appear to share common populations of neurons. The hypothesis of an increased brain excitability by backward adaptation is consistent with our recent MEG study^15^ demonstrating a power increase in the gamma oscillatory band in the attention network, and which persisted during a detection task performed after the adaptation exposure. Given that gamma oscillation power is known to increase in relation to the efficiency of sensory processing (e.g.^36–38^) this pattern of modulation, found by Nicolas et al.^15^, might contribute to the coupling between backward adaptation of RS and covert exogenous attention. Moreover, the role of the PPC in spatial representation in interaction with visuospatial attention has also been highlighted using prism adaptation (PA), as will be detailed below.

Importantly, the present study is the first to reveal that forward adaptation of leftward saccades led to an attentional boosting effect for targets in the right, but not in the left hemifield, providing new insights into the coupling between SA and attention. This result implies that the activity pattern or excitability of the cortical networks driving attention to the right hemifield was positively affected by forward SA. According to Corbetta et al.^39^, this cortical network should include the ventral system of the right hemisphere which supposedly encodes the entire visual space. However, Kim^40^ suggested in his recent meta-analysis that orienting attention to the right hemifield also relies on the left hemisphere. In this context, the effect of forward adaptation of leftward reactive saccades we found in the right hemifield suggests that forward adaptation involves activation of the left hemisphere.

Such involvement of the cerebral cortex (likely the PPC) of the left hemisphere may appear counter-intuitive if it is related to the adaptive change of the leftward (ipsiversive) saccadic vector. This movement vector coding hypothesis indeed predicts the same spatial pattern of attentional effect for a given direction of saccadic vector: for leftward reactive saccades studied here, both backward adaptation and forward adaptation should increase attention orienting in the left hemifield. Instead we found that the facilitation of attention orienting following backward adaptation or forward adaptation takes place respectively in the left or right hemifield. Since the only difference between the two adaptation conditions is the direction of the target jump, this pattern of results is consistent with an error signal hypothesis whereby the attention boosting effect results from a cortical activation related to the encoding of error signals which lead to adaptation.

Theoretical frameworks and empirical data from the prism adaptation literature are consistent with this line of thought. PA is another type of adaptation process elicited by a sensorimotor conflict, via the exposure to a prism-related shift of the visual field while individuals produce goal-directed reaching movements of the limb. Analogously to SA, PA can modulate attention orienting, as first demonstrated by an improvement of visuo-spatial perceptual tasks in left hemineglect patients^41–43^ and then as modifications of attentional performance in healthy subjects (see e.g.^44^). Also, the involvement of the PPC in PA has been disclosed by neuroimaging studies in healthy subjects and neglect patients^45–48^ suggesting that the cognitive effects of PA, notably the effects on visuo-spatial attention, rely on the PPC attentional system. The cerebellum has also long been known to be involved in PA^49,50^, a role classically assigned to the induction of plastic changes of motor output and to the generation of predictive signals.

In Pisella et al.’s^51^ model, the cerebellar hemisphere ipsilateral to the PA-induced visual deviation (visual error signal) is activated and inhibits the opposite PPC (contralateral to the visual deviation). In turn, through the release of interhemispheric inhibition between the two PPCs, this would result in dis-inhibition of the PPC ipsilateral to the PA-induced visual error. This inter-PPC imbalance would lead to an attentional orientation bias favoring the visual hemifield opposite the PA-induced visual error. This model is supported by a recent TMS-based investigation of cortical excitability in healthy subjects^52^. This study showed that, after leftward PA, the excitability of the left PPC-M1 tract increased, consistent with a trans-hemispheric dis-inhibition resulting from an inhibition of the right PPC.

As the PPC and cerebellum are also both involved in SA^4,6,15^, we speculate that this framework proposed for PA could also account for the presently demonstrated modifications of attention after adaptation of leftward reactive saccades. In the case of backward SA, the intra-saccadic step is rightward and, according to this framework, would activate the right cerebellum. Thus, the activation in the left PPC would decrease and that of the right PPC increase. This is consistent with the present improvement of attentional orienting toward the left hemifield. In the case of forward SA, the same reasoning would entail that, as the leftward intra-saccadic step would recruit the left cerebellum, the activation in the right PPC would decrease and that of the left PPC increase, yielding to the observed boost of attentional orienting toward the right hemifield. Also, according to this framework, we can speculate about backward adaptation of rightward saccades: the error signal arising from the left cerebellum would inhibit the right IPS and in turn, activate the left IPS. However, as the PPC of the left hemisphere is not likely to be involved in the capture of attention^39^, this increase of excitability following RS adaptation would not affect performance in an attentional task, as suggested by data published by Habchi et al.^14^.

To conclude, the error signal framework represents a promising hypothesis to account for the effects of SA on attention observed here. Further studies are necessary to provide a more comprehensive picture of the link between reactive saccade adaptation and exogenous attention orienting. Neurophysiological studies will have to decipher the cerebellar and cerebral underpinnings of this error vector hypothesis. Also, while the present behavioral study focused on leftward saccades, investigating saccades in the rightward direction would be an important follow-up study. This would test if this framework underlying the link between SA and attention is completely symmetrical relative to saccade direction, or if, as predicted from the findings of Habchi et al.^14^ in the reactive/exogenous modality and of Nicolas et al.^35^ in the voluntary/endogenous modality, is restricted to the leftward direction tested here.

Taken together, the present findings highlight a coupling between saccadic adaptation and visuospatial attention. This coupling could be explained by shared neuronal substrates at the level of the PPC. Our results further support the contribution of the motor system in the attention system and lead towards promising rehabilitation procedure for patients with visuospatial disorders.

## Supplementary information

Supplementary information

## Data Availability

Data will be made available by contacting the corresponding author.

## References

[1] Gaymard B, Ploner CJ, Rivaud S, Vermersch AI, Pierrot-Deseilligny C (1998). Cortical control of saccades Exp. Brain Res..

[2] Leigh RJ, Zee DS (1999). The Neurology of Eye Movements.

[3] Pélisson D, Alahyane N, Panouillères M, Tilikete C (2010). Sensorimotor adaptation of saccadic eye movements. Neurosci. Biobehav. Rev..

[4] Prsa M, Thier P (2011). The role of the cerebellum in saccadic adaptation as a window into neural mechanisms of motor learning: Role of the cerebellum in saccadic adaptation. Eur. J. Neurosci..

[5] Panouillères M (2014). A role for the parietal cortex in sensorimotor adaptation of saccades. Cereb. Cortex.

[6] Gerardin P, Miquée A, Urquizar C, Pélisson D (2012). Functional activation of the cerebral cortex related to sensorimotor adaptation of reactive and voluntary saccades. NeuroImage.

[7] Blurton SP, Raabe M, Greenlee MW (2012). Differential cortical activation during saccadic adaptation. J. Neurophysiol..

[8] Guillaume A, Fuller JR, Srimal R, Curtis CE (2018). Cortico-cerebellar network involved in saccade adaptation. J. Neurophysiol..

[9] Posner MI (1980). Orienting of attention. Q. J. Exp. Psychol..

[10] Carrasco M, Penpeci-Talgar C, Eckstein M (2000). Spatial covert attention increases contrast sensitivity across the CSF: Support for signal enhancement. Vision Res..

[11] Corbetta M, Shulman GL (2002). Control of goal-directed and stimulus-driven attention in the brain. Nat. Rev. Neurosci..

[12] Gerardin P, Nicolas J, Farnè A, Pélisson D (2015). Increasing attentional load boosts saccadic adaptationattention enhances oculomotor adaptation. Invest. Ophthalmol. Vis. Sci..

[13] McFadden SA, Khan A, Wallman J (2002). Gain adaptation of exogenous shifts of visual attention. Vision Res..

[14] Habchi O (2015). Deployment of spatial attention without moving the eyes is boosted by oculomotor adaptation. Front. Hum. Neurosci..

[15] Nicolas J (2019). Saccadic adaptation boosts ongoing gamma activity in a subsequent visuoattentional task. Cereb. Cortex.

[16] Ditterich J, Eggert T, Straube A (2000). Relation between the metrics of the presaccadic attention shift and of the saccade before and after saccadic adaptation. J. Neurophysiol..

[17] Khan A, McFadden SA, Harwood M, Wallman J (2014). Salient distractors can induce saccade adaptation. J. Ophthalmol..

[18] Schütz AC, Kerzel D, Souto D (2014). Saccadic adaptation induced by a perceptual task. J. Vis..

[19] Madelain L, Harwood MR, Herman JP, Wallman J (2010). Saccade adaptation is unhampered by distractors. J. Vis..

[20] Meermeier A, Gremmler S, Richert K, Eckermann T, Lappe M (2017). The reward of seeing: Different types of visual reward and their ability to modify oculomotor learning. J. Vis..

[21] Panouillères M (2009). Behavioral Evidence of separate adaptation mechanisms controlling saccade amplitude lengthening and shortening. J. Neurophysiol..

[22] Faul F, Erdfelder E, Lang A-G, Buchner A (2007). G* Power 3: A flexible statistical power analysis program for the social, behavioral, and biomedical sciences. Behav. Res. Methods.

[23] Peirce JW (2008). Generating stimuli for neuroscience using PsychoPy. Front. Neuroinformatics.

[24] Alahyane N, Pélisson D (2005). Long-lasting modifications of saccadic eye movements following adaptation induced in the double-step target paradigm. Learn. Mem..

[25] McLaughlin SC (1967). Parametric adjustment in saccadic eye movements. Percept. Psychophys..

[26] Dalmaijer ES, Mathôt S, Van der Stigchel S (2014). PyGaze: An open-source, cross-platform toolbox for minimal-effort programming of eyetracking experiments. Behav. Res. Methods.

[27] Chica AB, Martín-Arévalo E, Botta F, Lupiáñez J (2014). The Spatial Orienting paradigm: How to design and interpret spatial attention experiments. Neurosci. Biobehav. Rev..

[28] Chica AB, Bartolomeo P, Lupiáñez J (2013). Two cognitive and neural systems for endogenous and exogenous spatial attention. Behav. Brain Res..

[29] Bidet-Caulet A, Bottemanne L, Fonteneau C, Giard M-H, Bertrand O (2015). Brain dynamics of distractibility: interaction between top-down and bottom-up mechanisms of auditory attention. Brain Topogr..

[30] Benjamini Y, Hochberg Y (1995). Controlling the false discovery rate: A Practical and powerful approach to multiple testing. J. R. Stat. Soc. Ser. B. Methodol..

[31] Mosteller F, Tukey JW (1977). Data Analysis and Regression: A Second Course in Statistics.

[32] Jordan H, Tipper SP (1998). Object-based inhibition of return in static displays. Psychon. Bull. Rev..

[33] Luo C, Lupiáñez J, Funes MJ, Fu X (2013). Reduction of the spatial stroop effect by peripheral cueing as a function of the presence/absence of placeholders. PLoS ONE.

[34] Petersen SE, Posner MI (2012). The attention system of the human brain: 20 Years after. Annu. Rev. Neurosci..

[35] Nicolas J, Bidet-Caulet A, Pélisson D (2019). Inducing oculomotor plasticity to disclose the functional link between voluntary saccades and endogenous attention deployed perifoveally. Sci. Rep..

[36] Womelsdorf T, Fries P, Mitra PP, Desimone R (2006). Gamma-band synchronization in visual cortex predicts speed of change detection. Nature.

[37] Hoogenboom N, Schoffelen J-M, Oostenveld R, Fries P (2010). Visually induced gamma-band activity predicts speed of change detection in humans. NeuroImage.

[38] Tallon-Baudry C, Bertrand O (1999). Oscillatory gamma activity in humans and its role in object representation. Trends Cogn. Sci..

[39] Corbetta M, Patel G, Shulman GL (2008). The reorienting system of the human brain: From environment to theory of mind. Neuron.

[40] Kim H (2014). Involvement of the dorsal and ventral attention networks in oddball stimulus processing: A meta-analysis: Oddball stimulus processing. Hum. Brain Mapp..

[41] Rossetti Y (1998). Prism adaptation to a rightward optical deviation rehabilitates left hemispatial neglect. Nature.

[42] Striemer C, Danckert J (2007). Prism adaptation reduces the disengage deficit in right brain damage patients. NeuroReport.

[43] Nijboer TCW, McIntosh RD, Nys GMS, Dijkerman HC, Milner AD (2008). Prism adaptation improves voluntary but not automatic orienting in neglect. NeuroReport.

[44] Michel C (2006). Simulating unilateral neglect in normals: Myth or reality?. Restor. Neurol. Neurosci..

[45] Clower DM (1996). Role of posterior parietal cortex in the recalibration of visually guided reaching. Nature.

[46] Luauté J, Halligan P, Rode G, Rossetti Y, Boisson D (2006). Visuo-spatial neglect: A systematic review of current interventions and their effectiveness. Neurosci. Biobehav. Rev..

[47] Danckert J, Ferber S, Goodale MA (2008). Direct effects of prismatic lenses on visuomotor control: An event-related functional MRI study. Eur. J. Neurosci..

[48] Saj A, Cojan Y, Vocat R, Luauté J, Vuilleumier P (2013). Prism adaptation enhances activity of intact fronto-parietal areas in both hemispheres in neglect patients. Cortex.

[49] Weiner MJ, Hallett M, Funkenstein HH (1983). Adaptation to lateral displacement of vision in patients with lesions of the central nervous system. Neurology.

[50] Martin TA, Keating JG, Goodkin HP, Bastian AJ, Thach WT (1996). Throwing while looking through prisms: I. Focal olivocerebellar lesions impair adaptation. Brain.

[51] Pisella L (2005). Ipsidirectional impairment of prism adaptation after unilateral lesion of anterior cerebellum. Neurology.

[52] Martín-Arévalo E, Schintu S, Farnè A, Pisella L, Reilly KT (2016). Adaptation to leftward shifting prisms alters motor interhemispheric inhibition. Cereb. Cortex.

