# Supplementary material for: Reactive saccade adaptation boosts orienting of visuospatial attention
Source: Sci Rep. 2020 Aug 10;10:13430. doi: 10.1038/s41598-020-70120-z (PMC7417993; doi:10.1038/s41598-020-70120-z)
Supplement: Supplementary file 1 — Supplementary information [file 41598_2020_70120_MOESM1_ESM.docx]

“REACTIVE SACCADE ADAPTATION BOOSTS ORIENTING OF VISUOSPATIAL ATTENTION”

# AUTHORS

**Judith Nicolas^1,2,3^, Aurélie Bidet-Caulet^2^, and Denis Pélisson^1^**

1. Integrative Multisensory Perception Action & Cognition Team (ImpAct), INSERM U1028, CNRS UMR5292, Lyon Neuroscience Research Center (CRNL), 69000 Lyon, France
2. Brain Dynamics and Cognition (Dycog Team), INSERM U1028, CNRS UMR5292, Lyon Neuroscience Research Center (CRNL), 69000 Lyon, France
3. Present address: Movement Control and Neuroplasticity Research Group, Department of Kinesiology, KU Leuven, Leuven, Belgium

Corresponding author: **Judith Nicolas**. Movement Control & Neuroplasticity Research Group, Tervuursevest 101 - box 1501, 3001 Leuven, Belgium; [nicolasjdh@gmail.com](mailto:nicolasjdh@gmail.com); + 33 (0)6 65 77 47 46.

# APPENDIX

SUPPLEMENTARY METHODS

Power analysis. The number of subjects was determined from the following power analysis performed through the G*Power software ^1^. As stated in Introduction, we are aware of only one previous study testing the coupling between SA and visuospatial attention in the reactive/exogenous modality ^2^. This study disclosed that after adaptation of reactive saccades in the left hemifield, the performance in a visual detection task improved in the left hemifield. This was revealed by a significant 3-level interaction in a repeated measures ANOVA (rmANOVA) with subjects as the repeated measure, the target hemifield (left or right), the phase (pre- or post-exposure) and the exposure (leftward adaptation, rightward adaptation, or control) as within-factors. We have computed the effect size of this interaction and found a value of 0.41 (Sum of Square of the numerator = 485.7; Sum of Square of the denominator = 695.8), resulting in an achieved power larger than 99% according to the G*Power software. However, since the dependent variable in the present study is not the same (cue benefit *vs* reaction time), we decided to reduce this effect size to a conservative level of 0.2.

Given this medium effect size (*f* = 0.2), we found that 14 subjects are required for our interaction of interest between the 3 within-subjects to reach a power of 95.3% when assuming an average correlation coefficient of *r* = 0.85 between repeated measures (from our own pilot data) and a nonsphericity correction of *ε*=0.5. To counterbalance the six possible testing orders in the sessions included in our design, we decided to increase this number up to 18 subjects, reaching a power of 98.9%.

Instructions and feedback. Before the experiment started, the experimenter first displayed on the screen the two types of trials (informative and uninformative) and informed subjects about the presence of ‘No-Target’ trials. Then, the experimenter displayed instructions on how feedback about their detection performances will be provided after each block of trials, namely by means of a gauge filling up or down depending on subject’s performance (translated from French: “if you use the cue well enough to be fast, the gauge will fill up otherwise it will empty. The gauge will also empty if you answered to too many ‘No-Target’ trials.”). At the beginning of each session, this 10-graduation gauge was initially filled to the fourth graduation. At the end of each block the gauge was presented filled up to a new graduation according to the subjects’ score during the block: one graduation was gained if they were faster in informative trials than in uninformative trials, and two graduations were gained when the subjects additionally did not answer to more than 2 ‘No-Target’ trials. Conversely, one graduation was lost if subjects’ median performance was slower in informative trials than in uninformative trials. Increases or decreases of gauge levels were emphasized by a green or red filling color, respectively. In addition, each possible change of gauge level was accompanied by the following sentences: “Be careful, you need to better use the cue” (one graduation down), “Good, but you still can use the cue better” (one graduation up); “Bravo, keep on using the cue this way!” (two graduations up). Finally, a sentence was displayed requesting the subject to signal when she/he was ready to start the next block. These written feedbacks and instructions are intended to avoid non-standardized oral feedback from the experimenter who could not be blind regarding the type of session (backward, forward, or control exposure conditions).

SUPPLEMENTARY RESULTS

In order to account for reaction times (RT) variability and collapsed factors such as Block and Target eccentricity, we performed another analytic approach consisting of Generalized linear mixed-effects models (GLMM).

Indeed, generalized linear mixed-effects models (GLMM) are a good way to deal with the issue of variance of a variable of interest, as they allow for correction of systematic variability (Bates et al., 2014). We accounted for the heterogeneity of reaction times between subjects and experimental conditions by defining these factors as effects with a random intercept and slope, thus instructing the model to correct for any systematic difference in variability between the subjects (between-individual variability) and conditions (between-condition variability). To confirm the need for mixed nested models, we used a likelihood ratio analysis to test the model fit before and after sequential addition of random effects. We used the Akaike Information Criterion (AIC) and the Bayesian Information Criterion (BIC) as estimators of the quality of the statistical models generated ^3^. To optimize our model, we checked the normality of the model residuals. We ran a type II analysis of variance. Wald chi-square tests were used for ﬁxed effects in linear mixed-effects models ^4^. The fixed effect represents the mean effect across all subjects after correction of variabilities. Frequentist models and statistics were performed in R® 3.4.1 using the lme4^5^ and car^6^ packages. Main analyses were followed by post-hoc Honest Significant Difference (HSD) tests, using the emmeans package (emmeans version 1.3.2). P-values were considered as signiﬁcant at p < .05.

Raw RTs of every single trial were fitted to a linear model with a Gaussian family distribution (Model 1 in Supplementary table 1). The fixed factors were Exposure (backward, forward, and control), Phase (pre and post), Hemifield (left and right), and Validity (informative and uninformative). This first model accounted only for the interindividual variability (subject as intercept). We compared this simple model with an identical one for which raw RTs were log-transformed at the single trial scale (Model 2 in Supplementary table 1). Then we added the random effect Target eccentricity nested into the subject random effect (Model 3 in Supplementary table 1). Finally, we added the random effect Block nested into the subject random effect (Model 4 in Supplementary table 1). The results of the statistical comparisons of these models are reported in Supplementary table 2. The best model was Model 4 which accounted not only for the interindividual variability (subject as intercept) but also for the heterogeneity across subjects of the block effect and of the target eccentricity effect (slope).

Using Model 4, we found a significant three-way interaction involving the factors Exposure, Phase, and Hemifield (Wald chi-square = 6.84; df = 2; p = 0.033), as well as a three-way interaction involving the factors Exposure, Phase, and Validity (Wald chi-square = 7.94; df = 2; p = 0.019). The results of the post-hoc tests on raw RTs are summarized Supplementary table 3 (the mean values of the difference in milliseconds between the pre- and the post-phase (median RT pre – median RT post) in the different conditions). Note that the significant 3-way interactions confirm the conclusions of the analysis reported in the main manuscript and where the Validity factor is collapsed by using the Cue benefit as the dependent variable.

Supplementary table 1: Akaike Information Criterion (AIC) and the Bayesian Information Criterion (BIC) as a function of the GLMM Models

| Model | Dependent Variable | Random factors | AIC | BIC |
| --- | --- | --- | --- | --- |
| Model 1 | RT | 1\|Subject | 291143 | 291356 |
| Model 2 | Log(RT) | 1\|Subject | -20083 | -19870 |
| Model 3 | Log(RT) | TargetEccentricity\|Subject | -20638 | -20138 |
| Model 4 | Log(RT) | TargetEccentricity\|Subject + Block\|Subject | -20739 | -20189 |

Supplementary table 2: P-values issued from the statistical comparisons of the different models

| Comparison | P-value |
| --- | --- |
| Model 1 vs Model 2 | < 2.2e-16 |
| Model 2 vs Model 3 | < 2.2e-16 |
| Model 3 vs Model 4 | < 2.2e-16 |

Supplementary table 3: Pre-Post differences of RT (mean +/-SD) across subjects. Positive (negative) values indicate an increase (decrease) of RT in the post-exposure relative to the pre-exposure.

|  | Left | | Right | |
| --- | --- | --- | --- | --- |
|  | Informative | Uninformative | Informative | Uninformative |
| Backward | 6.41 +/- 14.39** | -1.03 +/- 17.61 | 0.18 +/- 11.30 | 1.85 +/-17.91 |
| Forward | 7.08 +/- 20.82*** | 2.79 +/-30.58 | 13.30 +/- 24.86*** | 8.67 +/- 26.38*** |
| Control | 8.68 +/- 15.94*** | 13.47 +/-21.02*** | 5.77 +/- 17.46*** | 16.47 +/- 22.65*** |

***: p-values < 0.01; ***: p-values < 0.001 (Post-Hoc tests of the GLMM)*

Finally, we sought for an impact of saccadic adaptation on saccade latency. For each subject, we computed the median of saccade latency (difference between the time of the target appearance and the beginning of the saccade) separately for each hemifield (left and right), each phase (pre- and post-exposure), and each type of exposure (backward, forward, and control). We then ran a 3-way rmANOVA (Exposure x Phase x Hemifield) with the median latency as dependent variable and subject as repeated measure.

This analysis revealed a main effect of the Phase (F_(1,17)_ = 5.2; p = 0.036). All other main effects and interactions were not significant. As shown in Figure 1 below, subjects’ saccade latencies were longer in the pre-exposure saccadic task as compared to the post-exposure saccadic task independently of the hemifield (adapted or un-adapted) and the exposure condition. This result is consistent with the results reported in a previous paper investigating the impact of voluntary saccade adaptation on endogenous orienting of attention^10^.


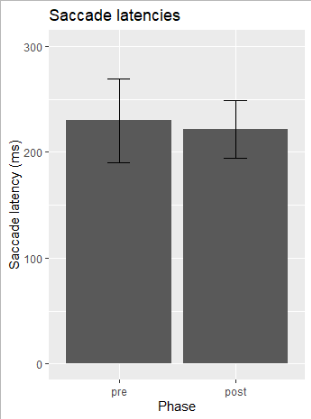


**Figure 1:** Group mean of median saccade latency (+/- SD) for pre- and post-exposure phases of the saccadic task.

**Supplementary references:**

1. Faul, F., Erdfelder, E., Lang, A.-G. & Buchner, A. G* Power 3: A flexible statistical power analysis program for the social, behavioral, and biomedical sciences. *Behav. Res. Methods* **39**, 175–191 (2007).

2. Habchi, O. *et al.* Deployment of spatial attention without moving the eyes is boosted by oculomotor adaptation. *Front. Hum. Neurosci.* **9**, (2015).

3. Matuschek, H., Kliegl, R., Vasishth, S., Baayen, H. & Bates, D. Balancing Type I error and power in linear mixed models. *J. Mem. Lang.* **94**, 305–315 (2017).

4. Fox, J. & Weisberg, S. Visualizing Fit and Lack of Fit in Complex Regression Models with Predictor Effect Plots and Partial Residuals. *J. Stat. Softw.* **87**, (2018).

5. Bates, D., Mächler, M., Bolker, B. & Walker, S. Fitting Linear Mixed-Effects Models Using lme4. *J. Stat. Softw.* **67**, (2015).

6. Fox, J. & Weisberg, S. *An R companion to applied regression*. (SAGE, 2019).
